# Cubical Sets and Trace Monoid Actions

**DOI:** 10.1155/2013/285071

**Published:** 2013-12-19

**Authors:** Ahmet A. Husainov

**Affiliations:** Faculty of Computer Technology, Komsomolsk-on-Amur State Technical University, Prospect Lenina 27, Komsomolsk-on-Amur 681013, Russia

## Abstract

This paper is devoted to connections between trace monoids and cubical sets. We prove that the category of trace monoids is isomorphic to the category of generalized tori and it is a reflective subcategory of the category of cubical sets. Adjoint functors between the categories of cubical sets and trace monoid actions are constructed. These functors carry independence preserving morphisms in the independence preserving morphisms. This allows us to build adjoint functors between the category of weak asynchronous systems and the category of higher dimensional automata.

## 1. Introduction

In this paper, it is established that the category of generalized tori is isomorpic to the category of trace monoids and basic homomorphisms. It is shown that the category of generalized tori is reflective subcategory of the category of cubical sets. Adjoint functors between the category of cubical sets and the category of trace monoids acting on sets are constructed. These functors carry independence preserving morphisms in the independence preserving morphisms. The results are used to compare asynchronous systems and higher dimensional automata for modelling of concurrent systems.

The problem of comparing the mathematical models of concurrent systems using adjoint functors has always been of great interest [1–5]. In [2], Goubault introduced automata with concurrency relations for generalization of asynchronous systems. The category ACR of automata with concurrency relations is isomorphic to the category of 2-skeletion of a category HTS of higher dimensional transition systems. The truncation gives the desired left adjoint to the inclusion functor ACR → HTS. Moreover, in the thesis [3], Goubault proved that there are adjoint functors between the category of semiregular higher dimensional automata and the category ATS of asynchronous transition systems satisfying the axiom of confluence.

But the asynchronous system

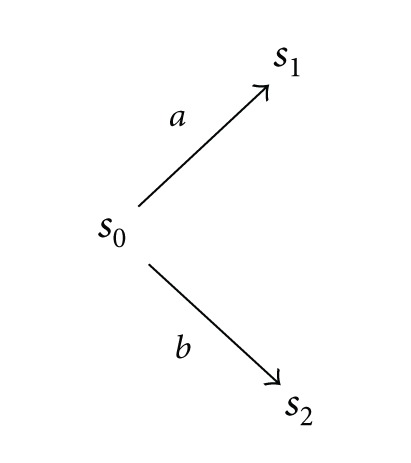
(1)
consisting of three states and two transitions with the independence relation *I* = {(*a*, *b*), (*b*, *a*)} does not satisfy the confluence condition and is not an automation with concurrency relation. Therefore, it remains an open problem of existence of a left adjoint functor from the category of higher dimensional automata in the category containing asynchronous systems which are not confluent.

This problem is solved in this paper.

We propose a category that includes all asynchronous systems and admits adjoint functors with the category of higher dimensional automata. By [6, 7], this category may be useful for studying the homology groups of asynchronous systems.

This work consists of two sections. In the first, we construct the adjoint functors between the categories of trace monoids and cubical sets. In the second, section we start with the construction of adjoint functors between the category of trace monoids acting on sets and the category of cubical sets. Then, we build adjoint functors between the categories of asynchronous systems and higher dimensional automata.

## 2. Trace Monoids and Cubical Sets

Introduce a category FPCM of trace monoids and basic homomorphisms. Consider a category of cubical sets. Introduce generalized tori. Construct the adjoint functors between the category FPCM of trace monoids and cubical sets.

### 2.1. Preliminaries

Throughout the paper, Set denotes the category of sets and maps.

For any category *𝒜* and objects *A*, *A*′ ∈ *𝒜*, let *𝒜*(*A*, *A*′) denotes the set of morphisms *f* : *A* → *A*′. Let *𝒜* ^op^ be the opposite category. A* diagram* (of objects) in *𝒜* is any functor from some small category *J* to *𝒜*. Let {*D*(*j*)}_*j*∈*J*_ denotes a diagram *D* : *J* → *𝒜*. *𝒜* ^*J*^ will always denote the category of diagrams *J* → *𝒜* and natural trasformations between them.

Let *S* : *𝒞* → *𝒟* be a functor between small categories and let *𝒜* be an arbitrary category. Consider the functor (−)∘*S* : *𝒜* ^*𝒟*^ → *𝒜* ^*𝒞*^ defined on objects as *F* ↦ *F*∘*S* and on morphisms as *η* ↦ *η*  ★  *S*. Left derived functor Lan^*S*^ : *𝒜* ^*𝒞*^ → *𝒜* ^*𝒟*^ to (−)∘*S* is called a *left Kan extension along S*.

Let *Y*′ : *𝒟* → Set^*𝒟*^op^^ be the Yoneda functor described in [8, III.2, page 62]. For each functor *H* : *𝒟* → *𝒜*, we consider the functor *D* : *𝒜* → Set^*𝒟*^op^^ defined on objects *A* ∈ *𝒜* by its values *D*(*A*)(−) = *𝒜*(*H*(−), *A*) with the obvious extension to morphisms.

**Proposition 1 (see [9, Proposition II.1.3])**

Let *𝒜* be a cocomplete category and let *𝒟* be a small category. For every functor *H* : *𝒟* → *𝒜* the functor *D* : *𝒜* → Set ^*𝒟*^*op*^^ has a left adjoint functor isomorphic to the left Kan extension *G* = Lan ^*Y*′^ *H*:

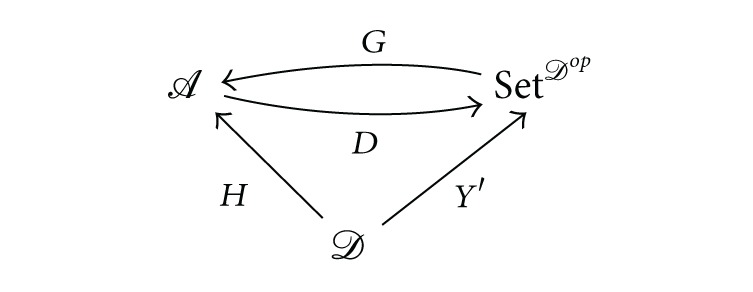
(2)

For any *X* ∈ Set ^*𝒟*^op^^, we have $\text{La}{\text{n}}^{Y^{\prime }}X={\underset{\to }{\lim}}^{𝒟↓X}(H\circ P_{X})$ where *𝒟* ↓ *X* = *Y*′ ↓ *X* denotes the comma category of objects *Y*′-over *X* for the Yoneda functor *Y*′ : *𝒟* → Set^□^op^^ and *P* = *P*_*X*_ : *𝒟* ↓ *X* → *𝒟* is the projection [8].

We can obtain by the theory of ends [8, Theorem X.4.1, page 240] the following.

**Proposition 2 (see [8])**

For each *X* ∈ Set ^*𝒟*^*op*^^, *G*(*X*) equals the colimit of a diagram defined on a directed bipartite graph consisting of morphisms

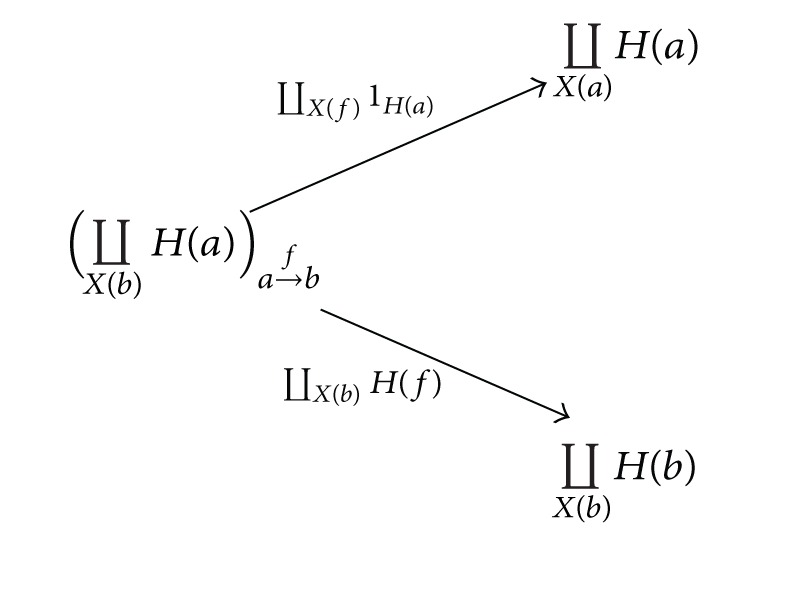
(3)
where *f* : *a* → *b* runs all morphisms of the category *𝒟*.

### 2.2. Trace Monoids

Let *E* be a set. A binary relation *I*⊆*E* × *E* is *irreflexive* if (∀*a* ∈ *E*) (*a*, *a*) ∉ *I*. One is *symmetric* if (∀(*a*, *b*) ∈ *E* × *E*) (*a*, *b*) ∈ *I*⇒(*b*, *a*) ∈ *I*. An *independence relation* on *E* is an arbitrary irreflexive symmetric binary relation *I*⊆*E* × *E*. In this case, elements *a*, *b* ∈ *E* are *independent* if (*a*, *b*) ∈ *I*.

Let E* be the set of all *words* *a*_1_ *a*_2_ ⋯ *a*_*n*_ composed of *letters a*_1_, *a*_2_,…, *a*_*n*_ ∈ *E* for all *n*⩾0. Then, E* is the monoid with the operation of *concatenation* by the following formula:

(4) $$\begin{array}{c} \left(a_{1}\cdots a_{n}\right)\left(b_{1}\cdots b_{m}\right)=a_{1}\cdots a_{n}b_{1}\cdots b_{m}. \end{array}$$

Identity element 1 is the empty word.

Let *I* be an independence relation on *E*. Define an equivalence relation ≡_*I*_ on E* assuming *w*_1_ ≡ *w*_2_ if the word *w*_2_ can be obtained from *w*_1_ by a finite sequence permutations of adjacent independent elements. For any *w* ∈ E*, its equivalence class [*w*] is called a *trace*. It is easy to see that the operation [*w*_1_][*w*_2_] = [*w*_1_ *w*_2_] transforms the set of equivalence classes E*/≡ in a monoid. This monoid is called a *trace monoid M*(*E*, *I*).

Our definition of trace monoid is different from that given in [10]. We suppose that *E* can be infinite.

In some cases, we omit square brackets and write *w* ∈ *M*(*E*, *I*) instead of [*w*] ∈ *M*(*E*, *I*). If *I* = *∅*, then *M*(*E*, *I*) is the free monoid E*. If *I* = ((*E* × *E*)∖{(*a*, *a*) | *a* ∈ *E*}), then *M*(*E*, *I*) is the *free commutative* monoid *ℕ* ^(*E*)^.

**Definition 3**

A homomorphism *f* : *M*(*E*, *I*) → *M*(*E*′, *I*′) is basic if *f*(*E*)⊆*E*′ ∪ {1}.

If *w* = *e*_1_ ⋯ *e*_*n*_ ∈ *M*(*E*, *I*) for some *e*_1_ ∈ *E*,…, *e*_*n*_ ∈ *E*, then *n* is the *length* of trace *w*.

**Proposition 4**

Every diagram *D* : *J* → *FPCM* has the colimit in *FPCM*.

**Proof**

Let *D*(*j*) = *M*(*E*_*j*_, *I*_*j*_) for *j* ∈ *J* and let ∐_*j*∈*J*_ *D*(*j*) be the coproduct in the category of monoids Mon. Then, the colimit ${\underset{\to }{\lim}}^{J}{\left\{D(j)\right\}}_{j\in J}$ is equal to the quotient monoid ∐_*j*∈*J*_ *M*(*E*_*j*_, *I*_*j*_)/≡_*I*_ where ≡_*I*_ is the smallest congruence relation satisfying $e\;\equiv_{I}\;D(i\overset{\alpha }{\to }j)(e)$ for all *i* ∈ *J*, *j* ∈ *J*, *α* ∈ *J*(*i*, *j*), *e* ∈ *E*_*i*_. A more detailed proof can be found in the preprint [11].

**Definition 5**

A basic homomorphism *f* : *M*(*E*, *I*) → *M*(*E*′, *I*′) is *independence preserving* if for all *a*, *b* ∈ *E* the following implication holds:

(5) $$\begin{array}{c} \left(a,b\right)\in I\Rightarrow \left(f\left(a\right)\neq f\left(b\right)\right)\vee \left(f\left(a\right)=f\left(b\right)=1\right). \end{array}$$

This is equivalent to the following condition:

(6) $$\begin{array}{c} \left(a,b\right)\in I\Rightarrow \left(f\left(a\right),f\left(b\right)\right)\in I^{\prime }\vee f\left(a\right)=1\vee f\left(b\right)=1. \end{array}$$

It follows that the composition of independence preserving homomorphisms is independence preserving. Let FPCM^||^ ⊂ FPCM be a subcategory consisting of all trace monoids and basic independence preserving homomorphisms.

### 2.3. Cubical Sets and Trace Monoids

A *cubical set *(*X*_*n*_, ∂_*i*_^*n*,*ν*^, *ϵ*_*i*_^*n*^) is a sequence of sets *X*_*n*_, *n* ∈ *ℕ* with two family of maps

- ∂_*i*_^*n*,*ν*^ : *X*_*n*_ → *X*_*n*−1_, 1 ⩽ *i* ⩽ *n*, *ν* ∈ {0,1}, (face operators)
- *ϵ*_*i*_^*n*^ : *X*_*n*_ → *X*_*n*+1_, 1 ⩽ *i* ⩽ *n* + 1, (degeneracies)

satisfying the following equations:

(7) $$\begin{array}{c} \partial_{i}^{n-1,\alpha }\partial_{j}^{n,\beta }=\partial_{j-1}^{n-1,\beta }\partial_{i}^{n,\alpha },\;2⩽i<j⩽n,\\ \epsilon_{i}^{n+1}\epsilon_{j}^{n}=\epsilon_{j+1}^{n+1}\epsilon_{i}^{n},\;1⩽i⩽j⩽n+1,\\ \partial_{i}^{n+1,\alpha }\epsilon_{j}^{n}=\{\begin{array}{cc} \epsilon_{j-1}^{n-1}\partial_{i}^{n,\alpha }, & 1⩽i<j⩽n+1,\\ \epsilon_{j}^{n-1}\partial_{i-1}^{n,\alpha }, & 1⩽j<i⩽n+1,\\ 1_{X_{n}}, & i=j. \end{array} \end{array}$$

A *morphism f* : *X* → *Y of cubical sets* is a family of maps *f*_*n*_ : *X*_*n*_ → *Y*_*n*_ commuting with the face operators and degeneracies. Let Cube be a category of cubical sets and morphisms.

Let □ be the category consisting of the partially ordered sets *𝕀* ^*n*^ = {0,1}^*n*^ and maps admitting decompositions by the following maps:

(8) $$\begin{array}{c} \delta_{i}^{n,\nu }\left(x_{1},\ldots ,x_{n}\right)=\left(x_{1},\ldots ,x_{i-1},\nu ,x_{i},\ldots ,x_{n}\right),\\ \varepsilon_{i}^{n}\left(x_{1},\ldots ,x_{n}\right)=\left(x_{1},\ldots ,x_{i-1},x_{i+1},\ldots x_{n}\right). \end{array}$$

By setting *X*(*𝕀* ^*n*^) = *X*_*n*_, *X*(*δ*_*i*_^*n*,*ε*^) = ∂_*i*_^*n*,*ε*^, *X*(*ε*_*i*_^*n*^) = *ϵ*_*i*_^*n*^, we can consider every cubical set as functor □^op^ → Set. A morphism of cubical sets can be considered as natural transformations. We will identify the category Cube with Set^□^op^^.

Introduce generalized tori.

**Definition 6**

Let *M*(*E*, *I*) be a trace monoid. *Generalized torus TM*(*E*, *I*) is a cubical set consisting of sets *T*_0_ *M*(*E*, *I*) = {1} and

(9) TnM(E,I)={(e1,…,en) ∣ ei∈E∪{1}  ∀1⩽i⩽n,       eiej=ejei  ∀1⩽i<j⩽n}

for all *n*⩾1. The maps ∂_*i*_^*n*,*ν*^, *ϵ*_*i*_^*n*^ are defined by

(10) $$\begin{array}{c} \partial_{i}^{n,\nu }\left(e_{1},\ldots ,e_{n}\right)=\left(e_{1},\ldots ,e_{i-1},e_{i+1},\ldots ,e_{n}\right),\\ \epsilon_{i}^{n}\left(e_{1},\ldots ,e_{n}\right)=\left(e_{1},\ldots ,e_{i-1},1,e_{i},\ldots ,e_{n}\right). \end{array}$$

The map *M*(*E*, *I*) ↦ *TM*(*E*, *I*) extends to a functor *T* : FPCM → Set^□^op^^ which assigns to each basic homomorphism *f* : *M*(*E*, *I*) → *M*(*E*′, *I*′) a morphism of cubical sets given by a family of maps *T*_*n*_ *f* : *T*_*n*_ *M*(*E*, *I*) → *T*_*n*_ *M*(*E*′, *I*′) defined as

(11) $$\begin{array}{c} T_{n}f\left(e_{1},\ldots ,e_{n}\right)=\left(f\left(e_{1}\right),\ldots ,f\left(e_{n}\right)\right). \end{array}$$

And conversely, since every morphism of cubical sets commutes with face operators, any morphism *η*_*n*_ : *T*_*n*_ *M*(*E*, *I*) → *T*_*n*_ *M*(*E*′, *I*′) can be given by the maps (*e*_1_,…, *e*_*n*_)↦(*η*_1_(*e*_1_),…, *η*_1_(*e*_*n*_)) defined by some *η*_1_ : *E* → *E*′ ∪ {1}. So, it holds the following.

**Proposition 7**

The functor *T* : *FPCM* → Set ^□^*op*^^ is full and faithful.

We will prove the following.

**Theorem 8**

The functor *T* : *FPCM* → Set ^□^*op*^^ has a left adjoint *G* : Set ^□^*op*^^ → *FPCM*. For every cubical set *X*, the trace monoid *G*(*X*) can be given by the generator set *X*_1_/≡ by a smallest equivalence relation on *X*_1_ identifying ∂_*i*_^2,0^ *x*_2_ ≡ ∂_*i*_^2,1^ *x*_2_ for all *x*_2_ ∈ *X*_2_ and *i* ∈ {1,2}, with the following relations for the equivalence classes:

(12) $$\begin{array}{c} \left[\partial_{1}^{2,0}x_{2}\right]\left[\partial_{2}^{2,0}x_{2}\right]=\left[\partial_{2}^{2,0}x_{2}\right]\left[\partial_{1}^{2,0}x_{2}\right],\;\forall x_{2}\in X_{2},\\ \left[\epsilon_{1}^{0}\left(x_{0}\right)\right]=1,\;\forall x_{0}\in X_{0}. \end{array}$$

**Proof**

For the construction of left adjoint to *T*, we use Propositions 1 and 2. With this aim, define a functor *H* : □→FPCM by setting *H*(*𝕀* ^*n*^) = *ℕ* ^*n*^ on objects of □. Let *H*(*δ*_*i*_^*n*,*ν*^)(*a* ^*k*_1_^,…, *a* ^*k*_*n*_^) = (*a* ^*k*_1_^,…, *a* ^*k*_*i*−1_^, 1, *a* ^*k*_*i*_^,…, *a* ^*k*_*n*_^) for all 1 ⩽ *i* ⩽ *n* and *ε* ∈ {0,1}. Set *H*(*ε*_*i*_^*n*^) : *ℕ* ^*n*^ → *ℕ* ^*n*−1^ by

(13) $$\begin{array}{c} H\left(\varepsilon_{i}^{n}\right)\left(a^{k_{1}},\ldots ,a^{k_{n}}\right)=\left(a^{k_{1}},\ldots ,a^{k_{i-1}},a^{k_{i+1}},\ldots ,a^{k_{n}}\right). \end{array}$$

Here, *a* ^*k*^ ∈ *ℕ* are elements of the free monoid generated by one element *ℕ* = {1, *a*, *a* ^2^,…}. Let *a*_*i*_ ∈ *ℕ* ^*n*^ be the tuple (1,…, 1, *a*, 1,…, 1) in which the generator *a* is located on the *i*th place. In particular, *a*_1_ = (*a*, 1,…, 1).

Basic homomorphisms *ℕ* ^*n*^ → *M*(*E*, *I*) are given by values at *a*_*i*_, 1 ⩽ *i* ⩽ *n*. Therefore, FPCM(*ℕ* ^*n*^, *M*(*E*, *I*))≅*T*_*n*_ *M*(*E*, *I*). It follows that the functor *T* is isomorphic to *D* acting by *D*(*M*) = FPCM(*H*(−), *M*) : □^op^ → Set. Hence, the functor *T* has a left adjoint *G*.

By Proposition 2, the object *G*(*X*) is isomorphic to quotient monoid of ∐_*n*⩾0,*x*∈*X*_*n*__(*H*(*𝕀* ^*n*^))_*x*_ by the smallest congruence relation identificating the pairs:

(14)     (x,H(f)(u))≡(X(f)(x),u),∀f∈(𝕀m,𝕀n), u∈H(𝕀m), x∈Xn.

Element *u* ∈ *ℕ* ^*m*^ equals (*a* ^*k*_1_^,…, *a* ^*k*_*m*_^) for some nonnegative integers *k*_*i*_.

Substituting *f* by *δ*_*i*_^*n*−1,*ν*^ and *ε*_*i*_^*n*+1^ and using the commutativity of monoids {*x*} × *ℕ* ^*n*^, we conclude that monoid *G*(*X*) can be given by the generators (*x*, *a*_*j*_) with the relations

(15) $$\begin{array}{c} \left(x,a_{i}\right)\left(x,a_{j}\right)\equiv \left(x,a_{j}\right)\left(x,a_{i}\right),\;1⩽i<j⩽n,\; \end{array}$$

(16) $$\begin{array}{c} \left(\partial_{i}^{n,\nu }\left(x\right),a_{j}\right)\equiv \{\begin{array}{cc} \left(x,a_{j}\right), & 1⩽j<i⩽n,\\ \left(x,a_{j+1}\right), & 1⩽i⩽j⩽n-1, \end{array} \end{array}$$

(17) $$\begin{array}{c} \left(\partial_{i}^{n,\nu }\left(x\right),1\right)=\left(x,1\right),\;1⩽i⩽n,\;\;\nu \in \left\{0,1\right\}, \end{array}$$

(18) $$\begin{array}{c} \left(\epsilon_{i}^{n}\left(x\right),a_{j}\right)\equiv \{\begin{array}{cc} \left(x,a_{j-1}\right), & 1⩽i<j⩽n+1,\\ \left(x,1\right), & 1⩽i=j⩽n+1,\\ \left(x,a_{j}\right), & 1⩽j<i⩽n+1. \end{array} \end{array}$$

The relation (15) follows from commutativity of {*x*} × *ℕ* ^*n*^. The relations (16) are obtained by the identifications:

(19) (∂in,ν(x),u)≡(x,H(δin,ν)(u)), ∀n⩾1,  1⩽i⩽n,           ν∈{0,1}, x∈Xn, u∈H(𝕀n−1).

The relations (18) can be obtained from

(20) $$\begin{array}{c} \left(x,H\left(\varepsilon_{i}^{n+1}\right)u\right)\equiv \left(\epsilon_{i}^{n}\left(x\right),u\right),\;\forall x\in X_{n},\;\;u\in \;{\mathbb{N}}^{n+1} \end{array}$$

by the substitution *u* = *a*_*j*_.

Identity (neutral) elements in ∐_*n*⩾0_∐_*x*∈*X*_*n*__(*H*(*𝕀* ^*n*^))_*x*_ are equal. It follows that (*x*, 1) ≡ 1 for all *n*⩾0 and *x* ∈ *X*_*n*_.

Hence, the relations (17) can be removed leaving the (*x*, 1) ≡ 1.

It follows from (*x*, *a*_*n*_)≡(∂_*n*−1_^*n*,*ν*^ *x*, *a*_*n*−1_)≡⋯≡(∂_1_^2,*ν*^ ⋯ ∂_*n*−2_^*n*−1,*ν*^∂_*n*−1_^*n*,*ν*^(*x*), *a*_1_) that each generator equals (*γ*, *a*_1_) for some *γ* ∈ *X*_1_. Moreover for dim⁡(*x*) = *n*, for every 1 ⩽ *j* ⩽ *n*, the similar sequence of relations leads to

(21) $$\begin{array}{c} \left(x,a_{j}\right)\equiv \left(\partial_{1}^{2,\nu }\cdots \partial_{n-2}^{n-1,\nu }\partial_{n-1}^{n,\nu }\left(x\right),a_{1}\right). \end{array}$$

Consequently, it is enough to leave the generators corresponding to 1-cubes. All relations can be obtained by the relations between those generators corresponding to 1-cubes.

It is easy to see that, among the relations (18), can be left only the following identifications (for *i* = *j* = 1 and *n* = 0):

(22) $$\begin{array}{c} \left(x,1\right)\equiv \left(\epsilon_{1}^{0}\left(x\right),a_{1}\right),\;\forall x\in X_{0}. \end{array}$$

So, *G*(*X*) can be given by generators (*x*, *a*_1_), *x* ∈ *X*_1_. The class of (*ϵ*_1_^0^, *a*_1_) equals 1. Since (∂_2_^2,*ν*^(*x*), *a*_1_)≡(*x*, *a*_1_) and (∂_1_^2,*ν*^(*x*), *a*_1_)≡(*x*, *a*_2_), every 2-cube (23) gives (∂_2_^2,0^(*x*), *a*_1_)≡(∂_2_^2,1^(*x*), *a*_1_), (∂_1_^2,0^(*x*), *a*_1_)≡(∂_1_^2,1^(*x*), *a*_1_),

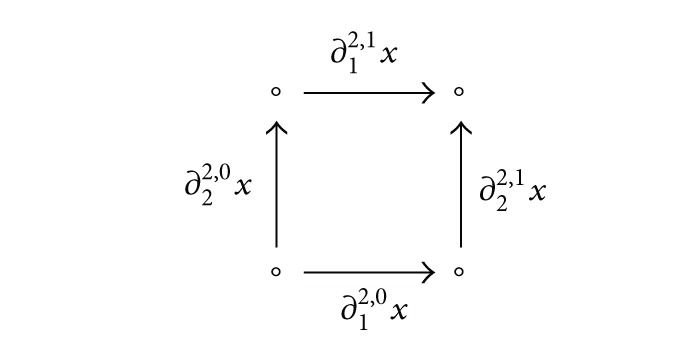
(23)

We have the following relations between the generators,

(24) (∂22,ν(x),a1)(∂12,ν(x),a1)  ≡(∂12,ν(x),a1)(∂22,ν(x),a1).

Since *a*_1_ = *a* for all pairs (*x*_1_, *a*_1_) with *x*_1_ ∈ *X*_1_, we can take the generators *x*_1_ ∈ *X*_1_ instead of generators (*x*_1_, *a*_1_). Thus, we obtain the desired generators and relations. And the class of *ϵ*_1_^0^(*x*) ∈ *X*_1_ equals 1.

For example, the cubical set shown in (23) has the monoid *G*(*X*) isomorphic to the free commutative monoid generated by two elements.

The category of generalized tori is the image of the functor *T*. By Proposition 7, that is, a full subcategory of Set^□^op^^ we have shown that *T* has the left adjoint *G*. By [8, Theorem IV.3.1], reflectivity is equivalent to that the counit of adjunction is an isomorphism. Therefore, we have the following.

**Corollary 9**

The subcategory of generalized tori is reflective in the category of cubical sets. In particular, counit of the adjunction *ε*_*M*(*E*,*I*)_ : *G* *TM*(*E*, *I*) → *M*(*E*, *I*) is an isomorphism.

### 2.4. Independence Preserving Morphisms

We introduce independence preserving morphisms of cubical sets. We prove that the category of cubical sets and independence preserving morphisms are linked with FPCM^||^ by adjoint functors.

**Definition 10**

A morphism of cubical sets *φ* : (*X*, ∂_*i*_^*n*,*ν*^, *ϵ*_*i*_^*n*^)→(*Y*, ∂_*i*_^*n*,*ν*^, *ϵ*_*i*_^*n*^) are *independence preserving* if

(25) (∀x∈X2), [∂12,0x]≠[∂22,0x],∂12,0x∉ϵ10(X0),∂22,0x∉ϵ10(X0) ⇒[∂12,0φ(x)]≠[∂22,0φ(x)]   ∨((∂12,0φ(x)∈ϵ10(Y0)),(∂22,0φ(x)∈ϵ10(Y0))).

Here, [*x*] denotes the congruence class of *x* ∈ *X*_1_ considered in Theorem 8.

Let Cube^||^ denotes the category of cubical sets and independence preserving morphisms. We construct the adjoint functors between FPCM^||^ and Cube^||^.  Definition 10 and Corollary 9 follow.

**Lemma 11**

Let *T* : *FPCM* → Set ^□^*op*^^ be the functor given in Definition 6 and let *G* : Set ^□^*op*^^ → *FPCM* be the left adjoint to *T*.

- A morphism of cubical sets *φ* : *X* → *Y* is independence preserving if and only if one is carried by the functor *G* to an independence preserving basic homomorphism *Gφ* : *G*(*X*) → *G*(*Y*).
- Basic homomorphism *f* : *M*(*E*, *I*) → *M*(*E*′, *I*′) is carried by the functor *T* to independence preserving morpism if and only if it is independence preserving.
- All components *ε*_*M*(*E*,*I*)_ : *G* *TM*(*E*, *I*) → *M*(*E*, *I*) of the counit are independence preserving.
- All components *η*_*X*_ : *X* → *TGX* of the unit are independence preserving.

**Proof**

The property (i) follows from Definition 10. Let us prove the last assertion. By Definition 10, it is equivalent to the statement that *G*(*η*_*X*_) : *G*(*X*) → *G*(*T*(*G*(*X*))) is independence preserving. Since *G* is left adjoint to *T*, the composition $G(X)\overset{G(\eta_{X})}{\to }G(T(G(X)))\overset{\varepsilon_{GX}}{\to }G(X)$ is equal to the identity morphism. The morphism $G(T(G(X)))\overset{\varepsilon_{GX}}{\to }G(X)$ is isomorphism by Corollary 9. Consequently, *G*(*η*_*X*_) : *G*(*X*) → *G*(*T*(*G*(*X*))) is an isomorphism. Thus, *η*_*X*_ : *X* → *T*(*G*(*X*)) is independence preserving by (i).

We obtain by Theorem 8 and by Lemma 11 the following.

**Theorem 12**

Restrictions of the functors *G* and *T* on the independence preserving morphisms give the adjoint functors

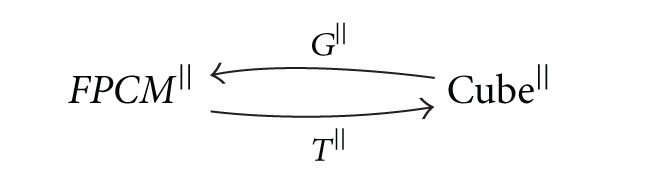
(26)

where *G* ^||^ is left adjoint to *T* ^||^.

## 3. Category of Trace Monoid Actions

We construct adjoint functors between a category of monoids acting on sets and the category of cubical sets.

### 3.1. Trace Monoid Actions

**Definition 13**

A trace monoid action (*M*(*E*, *I*), *X*) consists of a trace monoid *M*(*E*, *I*) with a set *X* and a map $X\times M(E,I)\overset{\cdot }{\to }X$ called a right action, (*x*, *μ*) ↦ *x* · *μ*, satysfying to the following conditions:

- (∀*x* ∈ *X*)  (∀*μ*_1_, *μ*_2_ ∈ *M*(*E*, *I*))  (*x* · *μ*_1_) · *μ*_2_ = *x* · (*μ*_1_ *μ*_2_),
- (∀*x* ∈ *X*)  *x* · 1 = *x*.

For example, for any trace monoid *M*(*E*, *I*), we have the trace monoid action (*M*(*E*, *I*), *M*(*E*, *I*)) defined as *x* · *μ* = *xμ* for all *x*, *μ* ∈ *M*(*E*, *I*). It is called the action *by right translations*.

**Definition 14**

A *morphism *(*f*, *σ*):(*M*(*E*, *I*), *X*)→(*M*(*E*′, *I*′), *X*′) of trace monoid actions consists of a homomorphism *f* : *M*(*E*, *I*) → *M*(*E*′, *I*′) and a map *σ* : *X* → *X*′ such those

(27) $$\begin{array}{c} \left(\forall x\in X\right)\;\;\left(\forall \mu \in M\left(E,I\right)\right)\;\sigma \left(x\cdot \mu \right)=\sigma \left(x\right)\cdot f\left(\mu \right). \end{array}$$

For example, for any homomorphism *f* : *M*(*E*, *I*) → *M*(*E*′, *I*′), the pair (*f*, *f*):(*M*(*E*, *I*), *M*(*E*, *I*))→(*M*(*E*′, *I*′), *M*(*E*′, *I*′)) is the morphism as satifying *f*(*x* · *μ*) = *f*(*x*)*f*(*μ*).

A morphism (*f*, *σ*):(*M*(*E*, *I*), *X*)→(*M*(*E*′, *I*′), *X*′) of trace monoid actions is *basic* if *f* is a basic homomorphism. It is called *independence preserving* if *f* is independence preserving.

Let (FPCM, Set) be a category of trace monoid actions and basic morphisms.

**Lemma 15**

The category (*FPCM*, Set) is cocomplete.

**Proof**

Monoid is a category with unique object. A monoid action (*M*, *X*) can be considered as a functor $\tilde{X}:M^{\text{op}}\to \text{Set}$ with the value *X* on the unique object of the category *M*. $\tilde{X}$ assigns to morphisms *μ* ∈ *M* the maps *X* → *X* such those $\tilde{X}(\mu )(x)=x\cdot \mu$. The following natural transformations $\tilde{\sigma }:\tilde{X}\to {\tilde{X}}^{\prime }\circ f^{\text{op}}$ corresponds to morphisms (*f*, *σ*):(*M*, *X*)→(*M*′, *X*′):

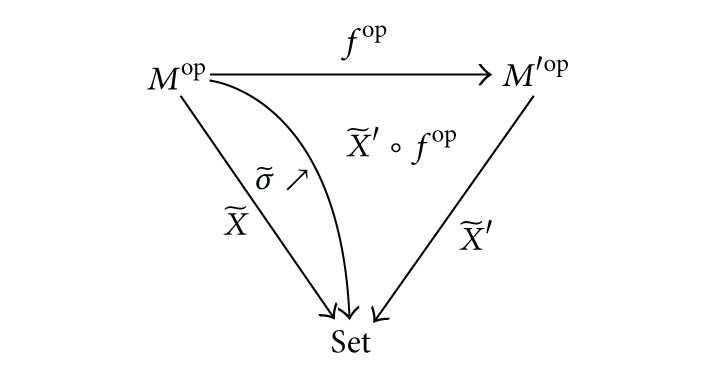
(28)

Consider an arbitrary diagram {(*M*(*E*_*i*_, *I*_*i*_), *X*_*i*_)}_*i*∈*J*_ in (FPCM, Set) as the diagram of functors ${\tilde{X}}_{i}:M{\left(E_{i},I_{i}\right)}^{\text{op}}\to \text{Set}$. There exists ${\underset{\to }{\lim}}^{J}\{M(E_{i},I_{i})\}$ in the category FPCM. Let {*λ*_*i*_}_*i*∈*J*_ be the colimit cone. Take left Kan extensions $\text{La}{\text{n}}^{\lambda_{i}^{\text{op}}}{\tilde{X}}_{i}$ belonging to the category of functors from ${\underset{\to }{\lim}}^{J}{\left\{M(E_{i},I_{i})\right\}}^{\text{op}}$ to Set. It is easy to see that desired colimit equals $\left({\underset{\to }{\lim}}^{J}\{M(E_{i},I_{i})\},{\underset{\to }{\lim}}^{J}\{\text{La}{\text{n}}^{\lambda_{i}^{\text{op}}}{\tilde{X}}_{i}\}\right)$.

We study colimits in the category (FPCM, Set) for diagrams of trace monoid actions by right translations. For an arbitrary small category, *J*, *π*_0_(*J*) denotes the set of connected components of *J*. For a set *S*, (*M*(*E*, *I*), *M*(*E*, *I*) × *S*) denotes a trace monoid with the action *by right translations *(*x*, *s*) · *μ* =_*def*_(*x* · *μ*, *s*) for all *x*, *μ* ∈ *M*(*E*, *I*), *s* ∈ *S*.

Let {*M*(*E*_*i*_, *I*_*i*_)}_*i*∈*J*_ be a diagram of trace monoids and basic homomorphisms *M*_*α*_ : *M*(*E*_*i*_, *I*_*i*_) → *M*(*E*_*j*_, *I*_*j*_) defined for all *i*, *j* ∈ Ob(*J*) and *α* ∈ *J*(*i*, *j*). Then, we have the diagram of trace monoid actions {(*M*(*E*_*i*_, *I*_*i*_), *M*(*E*_*i*_, *I*_*i*_))}_*i*∈*J*_ by right translations, consisting of morphisms (*M*_*α*_, *M*_*α*_).

**Proposition 16**

${\underset{\to }{\lim}}^{J}{\left\{(M(E_{i},I_{i}),M(E_{i},I_{i}))\right\}}_{i\in J}$ is isomorphic to the trace monoid action $({\underset{\to }{\lim}}^{J}\{M(E_{j},I_{j})\},\;{\underset{\to }{\lim}}^{J}\{M(E_{j},I_{j})\}\times \pi_{0}(J))\;$ by right translations.

**Proof**

*M*_*i*_ will denote *M*(*E*_*i*_, *I*_*i*_). Let $\lambda_{i}:M_{i}\to {\underset{\to }{\lim}}^{J}{\left\{M_{i}\right\}}_{i\in I}$ be the colimit cone. We prove the universality of the cone of morphisms $\left(M_{i},M_{i})\overset{\left(\lambda_{i},(\lambda_{i},\text{cls}(i)\right)}{\to }({\underset{\to }{\lim}}^{J}\{M_{i}\},{\underset{\to }{\lim}}^{J}\{M_{i}\}\times \pi_{0}(J)\right)$ defined as

(29) $$\begin{array}{c} \left(\lambda_{i},\left(\lambda_{i},\text{cls}\left(i\right)\right)\right)\left(\mu_{i},t_{i}\right)=\left(\lambda_{i}\left(\mu_{i}\right),\left(\lambda_{i}\left(t_{i}\right),\text{cls}\left(i\right)\right)\right)\; \end{array}$$

for all *μ*_*i*_, *t*_*i*_ ∈ *M*_*i*_. Here, cls(*i*) is the connected components of *J* containing *i* ∈ Ob(*J*).

Consider an arbitrary cone $\left(M_{i},M_{i})\overset{\left(\nu_{i},\xi_{i}\right)}{\to }(M(E,I),X\right)$ and construct a morphism (*ν*, *ξ*) making the following commutative diagram in (FPCM, Set):

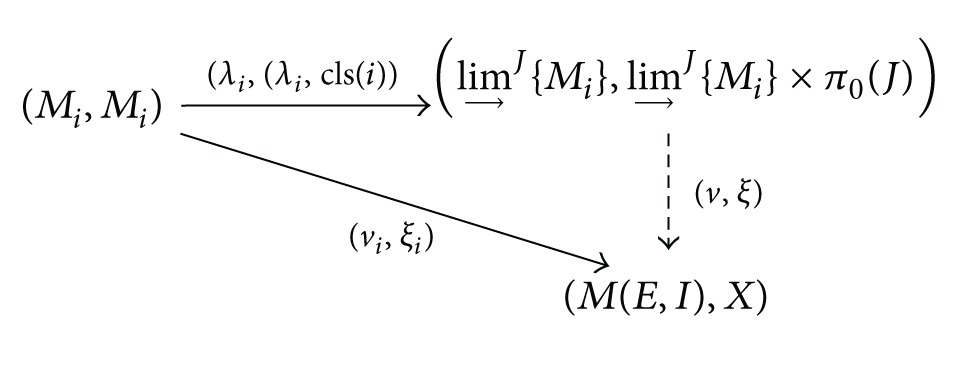
(30)
The existence and uniqueness of the homomorphism *ν* follow from the universal property of ${\underset{\to }{\lim}}^{J}\{M_{i}\}$ in FPCM.

Construct *ξ*. Since (*ν*_*i*_, *ξ*_*i*_) is cone, *ξ*_*i*_(1) = *ξ*_*j*_(1) for all *i*, *j* with same connected components. It follows that, for every *c* ∈ *π*_0_(*J*), the values *ξ*(1, *c*) = *ξ*_*i*_(1) must be determined by an arbitrary object *i* belonging to connected component *c*. These values give the unique extension *ξ*(*μ*, *c*) = *ξ*((1, *c*) · *μ*) = *ξ*(1, *c*) · *ν*(*μ*) to the set ${\underset{\to }{\lim}}^{J}\{M_{i}\}\times \pi_{0}(J)$. It follows the uniqueness of *ξ*. The equality of values *ξ*(*λ*_*i*_, cls(*i*))(1) = *ξ*_*i*_(1) at 1 ∈ *M*_*i*_ gives coincidence of compositions *ξ*∘(*λ*_*i*_, cls(*i*)) = *ξ*_*i*_ for all *i* ∈ Ob(*J*) and with it is the commutative triangles (30).

Further, let *G* : Set^□^op^^ → FPCM, *T* : FPCM → Set^□^op^^, *H* : □→FPCM denote the functors involved in Theorem 8. Let *Q* : (FPCM, Set) → Set^□^op^^ be the functor assigning to every trace monoid action (*M*(*E*, *I*), *S*) the cubical set

(31) Qn(M(E,I),S)={(s,e1,…,en):s∈S,  (∀i∈{1,…,n})  ei∈E∪{1},  eiej=ejei    ∀1⩽i<j⩽n}.

Recall that □↓*X* denotes the comma category *Y*′ ↓ *X* of objects *Y*′-over *X* (Proposition 1).

**Theorem 17**

The functor *Q* : (*FPCM*, Set) → Set ^□^*op*^^ has a left adjoint *G*′ : Set ^□^*op*^^ → (*FPCM*, Set) such that *G*′(*X*) = (*G*(*X*), *G*(*X*) × *π*_0_(□↓*X*)) are the trace monoid actions by right translation for all cubical sets *X*.

**Proof**

Let *H*′ : □→(FPCM, Set) be a functor defined as *H*′(*𝕀* ^*n*^) = (*ℕ* ^*n*^, *ℕ* ^*n*^) (with actions by right translations) at objects and *H*′(*f*) = (*H*(*f*), *H*(*f*)) for morphisms *f* ∈ Mor(□). The category (FPCM, Set) is cocomplete. Hence, this functor fits into the diagram

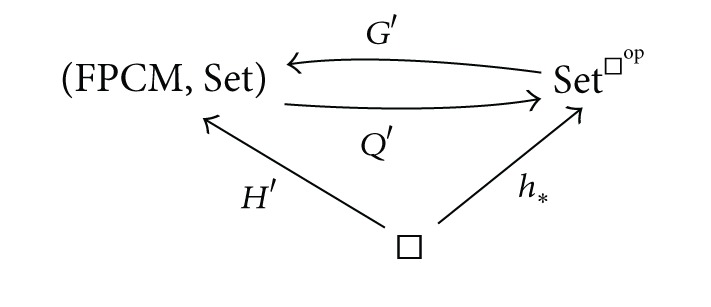
(32)
where *G*′ is left adjoint to *Q*′ taking values

(33) Q′(M(E,I),S)(𝕀n)  =(FPCM,Set)(H′(𝕀n),(M(E,I),S)).

Elements (*f*, *σ*)∈(FPCM, Set)(*H*′(*𝕀* ^*n*^), (*M*(*E*, *I*), *S*)) are determined by values *σ*(1) ∈ *S* where 1 is identity element of the monoid *ℕ* ^*n*^ and by the values of *f* on generators of the monoid *ℕ* ^*n*^. It follows that the functors *Q*′ and *Q* are isomorphic. For *X* ∈ Set^□^op^^, *G*′(*X*) is the colimit of a diagram consisting of trace monoids with actions by right translations (*H*(*𝕀* ^*n*^), *H*(*𝕀* ^*n*^)). The colimit in the first component equals *G*(*X*) by Theorem 8. We obtain *G*′(*X*) = (*G*(*X*), *G*(*X*) × *π*_0_(□↓*X*)) by Proposition 16.

### 3.2. Independent Morphisms of Trace Monoid Actions

By Lemma 11, a morphism of cubical set *φ* : *X* → *Y* is independence preserving if and only if the homomorphism of monoids *Gφ* : *GX* → *GY* is independence preserving.

**Definition 18**

A morphism (*f*, *σ*):((*M*(*E*, *I*), *S*)→(*M*(*E*′, *I*′), *S*′)) of the category (FPCM, Set) is independence preserving if *f* : *M*(*E*, *I*) → *M*(*E*′, *I*′) is independence preserving homomorphism.

Since *G*′*X* = (*GX*, *GX* × *π*_0_(□↓*X*)), it follows from this definition that *φ* : *X* → *Y* is independence preserving if and only if *G*′*φ* : *G*′*X* → *G*′*Y* is independence preserving. So, the functor *G*′ takes independence preserving morphisms into independence preserving.

The same is true for *Q*.

**Lemma 19**

The functor *Q* : (*FPCM*, Set ) → Set ^□^*op*^^ takes independence preserving morphisms into independence preserving.

**Proof**

By definition, a morphism *Q*(*f*, *σ*) : *Q*(*M*(*E*, *I*), *S*) → *Q*(*M*(*E*′, *I*′), *S*′) is independence preserving if and only if the homomorphism of monoids *GQ*(*f*, *σ*) : *GQ*(*M*(*E*, *I*), *S*) → *GQ*(*M*(*E*′, *I*′), *S*′) is independence preserving. The monoid *GQ*(*M*(*E*, *I*), *S*) is generated by classes [(*s*, *e*_1_)] such that [(*s*, *e*_1_)] = [(*s* · *e*, *e*_1_)] if (*e*, *e*_1_) ∈ *I*. We have also *ϵ*_1_^0^(*s*)≡(*s*, 1). Therefore, [(*s*, 1)] = 1. It follows that [(*s*, *e*)] = 1 if and only if *e* = 1. We need to prove the following implication:

(34) [(s,e1)][(s,e2)]=[(s,e2)][(s,e1)],[(s,e1)]≠[(s,e2)], [(s,e1)]≠1,[(s,e2)]≠1⇒[(σ(s),f(e1))]≠[(σ(s),f(e2))]∨([(σ(s),f(e1))]=[(σ(s),f(e2))]=1).

By the equivalences [(*s*, *e*)] ≡ 1⇔*e* = 1 in *GQ* *M*(*E*, *I*) and [(*s*, *e*_1_)]≠[(*s*, *e*_2_)]⇔*e*_1_ ≠ *e*_2_ in *M*(*E*, *I*), the premise of implication (34) is transformed into formula

(35) [(s,e1)][(s,e2)]=[(s,e2)][(s,e1)],       e1≠e2,  e1≠1,  e2≠1.  

Since the homomorphism *f* is independence preserving, we obtain the formula *f*(*e*_1_) ≠ *f*(*e*_2_)∨*f*(*e*_1_) = *f*(*e*_2_) = 1 leading to the conclusion of (34).

**Lemma 20**

The unit of the adjunction *η*_*X*_ : *X* → *QG*′*X* is independence preserving.

**Proof**

Since *G*′ is left adjoint to *Q*, the morphism $G^{\prime }X\overset{G^{\prime }(\eta_{X})}{\to }G^{\prime }QG^{\prime }X$ is a coretraction. It follows that the homomorphism of monoids $GX\overset{G(\eta_{X})}{\to }GQG^{\prime }X$ is a coretraction. It takes independent elements in the not equal. Hence, $GX\overset{G(\eta_{X})}{\to }GQG^{\prime }X$ is dependence preserving.

Let *ε*_(*M*(*E*,*I*),*S*)_ : *G*′*Q*(*M*(*E*, *I*), *S*)→(*M*(*E*, *I*), *S*) be the counit of adjunction *G*′⊣*Q*.

**Lemma 21**

The counit of adjunction *ε*_(*M*(*E*,*I*),*S*)_ is independence preserving.

**Proof**

The morphism *ε*_(*M*(*E*,*I*),*S*)_ consists of some pair (*f*, *σ*). It is independence preserving if and only if the homomorphism *f* : *GQ*(*M*(*E*, *I*), *S*) → *M*(*E*, *I*) is independence preserving. The monoid *GQ*(*M*(*E*, *I*), *S*) is generated by classes [(*s*, *e*)] of 1-cubes (*s*, *e*) ∈ *Q*_1_(*M*(*E*, *I*), *S*) = *S* × *E*. The homomorphism *f* assigns to each class [(*s*, *e*)] the element *e* ∈ *E*. If [(*s*, *e*_1_)]≠[(*s*, *e*_2_)] and ([(*s*, *e*_1_)], [(*s*, *e*_2_)]) are independed, then *e*_1_ ≠ *e*_2_. Hence, *f* is independence preserving.

It follows from obtained Lemmas.

**Theorem 22**

The restrictions of functors *Q* and *G*′ on the subcategories consisting of independence morphisms give adjoint functors

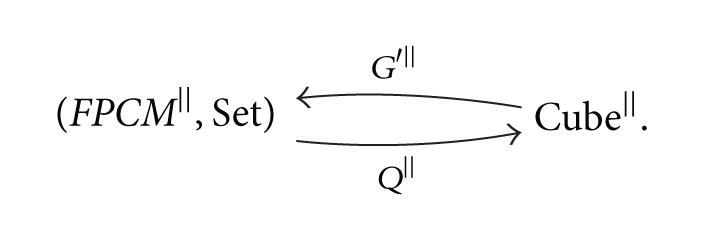
(36)

### 3.3. Asynchronous Systems and Higher Dimensional Automata

A *higher dimensional automation *(*X*, *x*_0_) consists of a cubical set *X* and an *initial point x*_0_ ∈ *X*_0_. A *morphism f* : (*X*, *x*_0_)→(*X*′, *x*_0_′)* of higher dimensional *automata is a morphism of cubical sets *f* : *X* → *X*′ such that *f*_0_(*x*_0_) = *x*_0_′. Let Cube_*p*_ be the category of higher dimensional automata and let Cube_*p*_^||^ be the category of higher dimensional automata and morphisms *f* : (*X*, *x*_0_)→(*X*′, *x*_0_′) for which *f* : *X* → *X*′ are independence preserving.

Let pt be the cubical set such that pt_*n*_ consists of unique element *p* for all *n*⩾0. Face operators and degeneracies are the identity maps.

It is easy to see that Cube_*p*_^||^≅pt ↓ Cube^||^.

Introduce a category of weak asynchronous systems. Consider partial maps of sets.

For any set *E*, let *E*_∗_ = *E*⊔{∗}. The set *E*_∗_ is called *pointed*. The element ∗ is the same for all pointed sets. A map *f* : *E*_1__∗_ → *E*_2__∗_ is *pointed* if *f*(∗) = ∗. Let Set_∗_ be the category of pointed sets and pointed maps.

Any partial map *f* : *E*_1_⇀*E*_2_ will be considered as the pointed map *f*_∗_ : *E*_1__∗_ → *E*_2__∗_ defined as follows:

(37) $$\begin{array}{c} f_{*}\left(a\right)=\{\begin{array}{cc} f\left(a\right), & \text{if}\;f\left(a\right)\;\text{is}\;\text{defined},\\ {}*, & \text{otherwise}.\; \end{array} \end{array}$$

This allows us to identify the category of sets and partial maps with the category Set_∗_.

A *(right) monoid action *(*M*, *S*_∗_)* on a pointed set* consists of a monoid *M* and a pointed set *S*_∗_ with an arbitrary map ·:*S*_∗_ × *M* → *S*_∗_ satisfying the following conditions:

- (∀*s* ∈ *S*_∗_)  (∀*μ*_1_, *μ*_2_ ∈ *M*)(*x* · *μ*_1_) · *μ*_2_ = *x* · (*μ*_1_ *μ*_2_),
- (∀*x* ∈ *S*_∗_)  *x* · 1 = *x*,
- (∀*μ* ∈ *M*)∗ ·  *μ* = ∗.

Let (*M*, *S*_∗_) be a monoid action on a pointed set. Since the monoid is a category with an unique object, (*M*, *S*_∗_) can be considered as a functor *M* ^op^ → Set_∗_ assigning to the unique object the pointed set *S*_∗_ and assigning to morphisms *μ* ∈ *M* the pointed maps (*M*, *S*_∗_)(*μ*) : *S*_∗_ → *S*_∗_ such those (*M*, *S*_∗_)(*μ*)(*x*) = *x* · *μ*.

It was shown in [12] that each asynchronous system can be considered as a trace monoid action on a pointed set with an initial point.

**Definition 23**

*A weak asynchronous system *(*M*(*E*, *I*), *S*_∗_, *s*_0_) is a trace monoid action (*M*(*E*, *I*), *S*_∗_) on a pointed set *S*_∗_ with an *initial point s*_0_ ∈ *S*_∗_.

If we require *S* ≠ *∅* and *s*_0_ ∈ *S*, then we get the definition of an asynchronous system in the sense of Bednarczyk [1].

A *polygonal morphism *(*f*, *σ*):(*M*(*E*, *I*), *S*_∗_, *s*_0_)→(*M*(*E*′, *I*′), *S*_∗_′, *s*_0_′) of weak asynchronous systems is an independence preserving basic homomorphism *f* : *M*(*E*, *I*) → *M*(*E*′, *I*′) with a pointed map *σ* : *S*_∗_ → *S*_∗_ satisfying the following conditions:

- (∀*s* ∈ *S*_∗_)  (∀*μ* ∈ *M*(*E*, *I*))  *σ*(*s* · *μ* = *σ*(*s*) · *f*(*μ*)),
- *σ*(*s*_0_) = *s*_0_′.

The map *f* is pointed from where *f*(∗) = ∗. Let *𝒜S* ^*♭*^ be the category of weak asynchronous systems and polygonal morphisms. Construct a functor *W* : *𝒜S* ^*♭*^ → Cube_*p*_^||^ assigning to every asynchronous system (*M*(*E*, *I*), *S*_∗_, *s*_0_) the cubical set consisting of a sequence of pointed sets

(38) Wn(M(E,I),S∗,s0)  ={(s,e1,…,en) ∣ s∈S∗, (e1,…,en)∈TnM(E,I)}

and *W*_0_(*M*(*E*, *I*), *S*_∗_, *s*_0_) = *S*_∗_. Face operators are defined as follows:

(39) $$\begin{array}{c} \partial_{i}^{n,\nu }\left(s,e_{1},\ldots ,e_{n}\right)=\left(s\cdot e_{i}^{\nu },e_{1},\ldots ,e_{i-1},e_{i+1},\ldots ,e_{n}\right). \end{array}$$

Degeneracies are defined by *ϵ*_*i*_^*n*^(*s*, *e*_1_,…, *e*_*n*_) = (*s*, *e*_1_,…, *e*_*i*−1_, 1, *e*_*i*_,…, *e*_*n*_). Initial point in *W*(*M*(*E*, *I*), *S*_∗_, *s*_0_) equals *s*_0_ ∈ *S*_∗_.

**Theorem 24**

The functor *W* : *𝒜S* ^*♭*^ → Cube _*p*_^||^ has a left adjoint *G*′′ : Cube _*p*_^||^ → *𝒜S* ^*♭*^.

**Proof**

Let *U* : Set_∗_ → Set be the functor forgetting the point ∗ and let *L* : Set → Set_∗_ be the left adjoint to *U* adding the point ∗. Let $\bar{U}=(\text{FPC}{\text{M}}^{||},U)$ be the functor (FPCM^||^, Set_∗_)→(FPCM^||^, Set) assigning to every trace monoid action *X* : *M*(*E*, *I*)^op^ → Set_∗_ the composition *U*∘*X* and assigning to morphisms $X\overset{\xi }{\to }X^{\prime }\circ f^{\text{op}}$ the morphisms *U*∗*ξ*. Similarly, we define the functor $\bar{L}:(\text{FPC}{\text{M}}^{||},\text{Set})\to (\text{FPC}{\text{M}}^{||},\text{Se}{\text{t}}_{*})$. Let *η* : Id_Set_ → *U*∘*L* be a unit and let *ε* : *L*∘*U* → Id_Set_∗__ be a counit of adjunction *L*⊣*U*. Since the functor *L* is left adjoint to *U*, the compositions $U\overset{\eta *U}{\to }U\circ L\circ U\overset{U*\varepsilon }{\to }U$ and $L\overset{L*\eta }{\to }L\circ U\circ L\overset{\varepsilon *L}{\to }L$ are equal to identity natural transformations. Construct natural transformations $\bar{\eta }:{\text{Id}}_{\left(\text{FPC}{\text{M}}^{||},\text{Set}\right)}\to \bar{U}\circ \bar{L}$ and $\bar{\varepsilon }:\bar{L}\circ \bar{U}\to {\text{Id}}_{\left(\text{FPC}{\text{M}}^{||},\text{Se}{\text{t}}_{*}\right)}$ as follows: ${\bar{\eta }}_{X}=\eta *X$, ${\bar{\varepsilon }}_{X}=\varepsilon *X$. Components of natural transformations $\bar{U}\overset{\bar{\eta }*\bar{U}}{\to }\bar{U}\circ \bar{L}\circ \bar{U}\overset{\bar{U}*\bar{\varepsilon }}{\to }\bar{U}$ and $\bar{L}\overset{\bar{L}*\bar{\eta }}{\to }\bar{L}\circ \bar{U}\circ \bar{L}\overset{\bar{\varepsilon }*\bar{L}}{\to }\bar{L}$ on objects equal identity natural transformations. Therefore, $\bar{L}$ is left adjoint to $\bar{U}$.

Consider the compositions

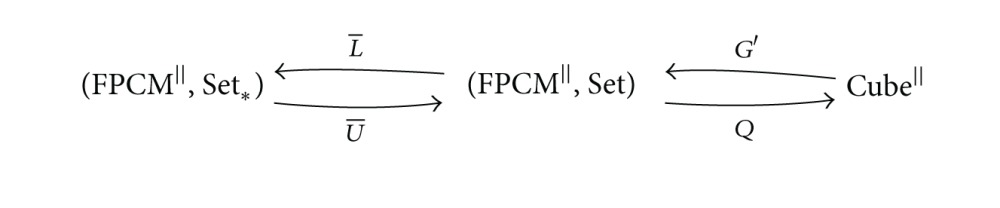
(40)
where *G*′ and *Q* are the adjoint functors from Theorem 22. The functor $\bar{L}\circ G^{\prime }$ is left adjoint to $Q\circ \bar{U}$.

The monoid {1} has the unique action on the set {*p*, ∗}. This trace monoid action equals $\bar{L}(G^{\prime }(\text{pt}))$. We have the adjoint functors between comma categories

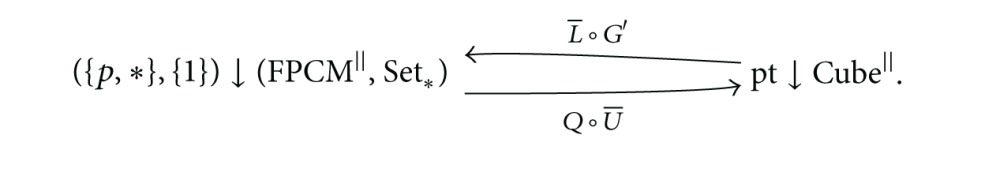
(41)

It easy to see that the category ({*p*, ∗}, {1})↓(FPCM^||^, Set_∗_) is isomorphic to *𝒜S* ^*♭*^. The composition of this isomorphism with $Q\circ \bar{U}$ is equal to the functor *W*. We obtain from this the functor $\bar{L}\circ G^{\prime }$ left adjoint to *W*.

## 4. Conclusion

We have considered the category of trace monoids and basic homomorphisms and proved that this category has all colimits. This allowed us to show that the category of generalized tori is a reflective subcategory of the category of cubical sets. Then, we considered the category of trace monoid actions and proved that it is cocomplete. We built adjoint functors between the category of trace monoid actions and the category of cubical sets. We unexpectedly found that these adjoint functors translate the independence preserving morphisms in independence preserving. As a result, we have completely solved the problem of comparing the category of asynchronous systems with the category of higher dimensional automata. Earlier, the problem had been solved only for asynchronous systems satisfying the confluence condition.

## References

[1] Bednarczyk M (1988). *Categories of asynchronous systems [Ph.D. thesis]*.

[2] Goubault E Labeled cubical sets and asynchronous transitions systems: an adjunction. http://www.lix.polytechnique.fr/~goubault/papers/cmcim02.ps.gz.

[3] Goubault E (1995). *The geometry of concurrency [Ph.D. thesis]*.

[4] Goubault E, Mimram S (2012). Formal relationships between geometrical and classical models for concurrency. *Electronic Notes in Theoretical Computer Science*.

[5] Winskel G, Nielsen M, Abramsky S, Gabbay M, Maibaum T (1995). Models for concurrency. *Handbook of Logic in Computer Scienceed*.

[6] Husainov AA (2012). Homology groups of asynchronous systems, Petri nets, and trace languages. *Siberian Electronic Mathematical Reports*.

[7] Husainov AA (2011). *The Cubical Homology of Trace Monoids*.

[8] Mac Line S (1998). *Categories for the Working Mathematishian, Graduate Texts in Mathematics*.

[9] Gabriel P, Zisman M (1967). *Calculus of Fractions and Homotopy Theory*.

[10] Diekert V, Metivier Y (1997). Partial commutation and traces. *Handbook of Formal Languages*.

[11] Husainov AA (2013). *Category of Asynchronous Systems and Polygonal Morphisms*.

[12] Husainov AA (2004). On the homology of small categories and asynchronous transition systems. *Homology, Homotopy and Applications*.

